# (Secondary) solid tumors in thyroid cancer patients treated with the multi-kinase inhibitor sorafenib may present diagnostic challenges

**DOI:** 10.1186/s12885-016-2060-4

**Published:** 2016-01-19

**Authors:** Tatiana C. Schneider, Ellen Kapiteijn, Tom van Wezel, Jan W. A. Smit, Jacobus J. M. van der Hoeven, Hans Morreau

**Affiliations:** Department of Clinical Oncology, Leiden University Medical Center, Leiden, The Netherlands; Department of Pathology, Leiden University Medical Center, Leiden, The Netherlands; Department of Endocrinology, Radboud University Medical Center, Nijmegen, The Netherlands

**Keywords:** Sorafenib, Differentiated thyroid cancer, Squamous cell carcinoma, Squamous differentiation, Clonal divergence

## Abstract

**Background:**

Sorafenib is an orally active multikinase tyrosine kinase inhibitor (TKI) that targets B-type Raf kinase (BRAF), vascular endothelial growth factor receptors (VEGFR) 1 and 2, and rearranged during transfection (RET), inducing anti-angiogenic and pro-apoptotic actions in a wide range of solid tumors. A side effect of sorafenib is the occurrence of cutaneous squamous tumors.

**Case presentation:**

Here we describe three patients with a history of sorafenib treatment for advanced radioactive iodine refractory papillary thyroid cancer (two with a *BRAF* c.1799 T > A and one carrying a rare c.1799-1801het_delTGA mutation) who presented with secondary non-cutaneous lesions. The first patient was diagnosed with a squamous cell carcinoma (SCC) of the tongue, the second patient with a primary adenocarcinoma of the lung, and the third with a SCC originating from the cricoid. Secondary analysis was required to show that the latter two presentations were in fact recurrent thyroid cancer.

**Conclusion:**

These findings suggest that drugs such as sorafenib may induce metaplasia/clonal divergence of metastatic thyroid cancer and thus cause diagnostic misclassification. Furthermore, sorafenib is potentially involved in the tumorigenesis of secondary non-cutaneous SCC. These observations should now be confirmed in larger series of patients treated with drugs such as sorafenib.

## Background

Sorafenib (BAY-43-9006) is an orally active multikinase tyrosine kinase inhibitor (TKI) that activates anti-angiogenic and pro-apoptotic pathways, targeting the *B-type Raf kinase* (BRAF), vascular endothelial growth factor receptors (VEGFR) 1 and 2, and *rearranged during transfection* (RET). Sorafenib is widely approved for the treatment of patients with hepatocellular carcinoma (HCC) and advanced renal cell carcinoma (RCC) in well-defined phases of disease. Since June 2015 sorafenib is also registered for the treatment of patients with thyroid cancer by the European Medicines Agency (EMA). Multiple clinical trials have been conducted in a wide range of cancers (lung, thyroid, breast, colorectal) using sorafenib as a single agent or in combination treatment (www.clinicaltrials.gov).

Recently published results of a phase III trial of sorafenib in patients with advanced radioactive iodine 131-I (RAI) refractory differentiated thyroid carcinoma (DTC) showed that sorafenib has clinically relevant antitumor activity and a generally well-tolerated profile of adverse events (AEs). The most commonly reported sorafenib-related AEs in DTC include hand-foot syndrome, hypertension, weight loss, diarrhea and rash [1, 2]. Recent reports have also suggested a possible causal link between sorafenib therapy and the development of cutaneous squamous cell carcinomas (SCC) [3–8].

We now describe three patients who received sorafenib during treatment for advanced RAI refractory DTC and presented with secondary non-cutaneous squamous lesions.

## Materials and methods

The presence of somatic DNA mutations including *BRAF* (V600E and V600K), *KRAS* (codon 12/13), and *PIK3CA* (exons 9 and 20) were determined by quantitative real-time PCR (qPCR) with hydrolysis probes (Custom TaqMan® Assay Design Tool, Applied Biosystems, Nieuwerkerk a/d IJssel, NL), and when indicated, by standard Sanger DNA sequencing on an ABI 3739 automated sequencer (Applied Biosystems, Foster City, CA, USA) [9]. Tissues were microdissected to enrich for tumor cells and tumor areas were selected based on the analysis of a hematoxylin eosin (HE)-stained tissue slide. Tumor DNA was subsequently isolated using the Nucleospin Tissue kit (Marcherey-Nagel, Bethlehem, PA, USA) according to manufacturer’s protocol.

### Case presentation 

#### Patient 1

A 67-year-old female was diagnosed with a well differentiated papillary thyroid carcinoma (PTC, *BRAF* c.1799 T > A; p.V600E mutation positive), stage T2N0M0, in 1989. She underwent a total thyroidectomy and RAI ablation therapy. Local recurrent disease was diagnosed ten years later and treated with a left-sided modified radical neck dissection, followed by RAI therapy. The post-therapy scintigraphy showed no RAI uptake. Six years later routine follow-up with computed tomography (CT) identified metastatic disease with multiple lung lesions, the largest measuring 5 mm (2005). A total body scintigraphy after RAI therapy showed no uptake of RAI despite elevated thyroglobulin levels (68 ug/l), indicating RAI refractory disease. Due to the progression of disease two years later (2007) the patient received sorafenib (2 x 400 mg/day initially, reduced to 1 x 200 mg/day after 6 months) in the context of a phase II study [2, 10]. Follow-up showed stable disease according to the Response Evaluation Criteria in Solid Tumours (RECIST) 1.0 [11]. Thirty-nine months after starting sorafenib, with ongoing stable disease, the patient stopped therapy mainly due to diarrhea.

**Fig. 1:**
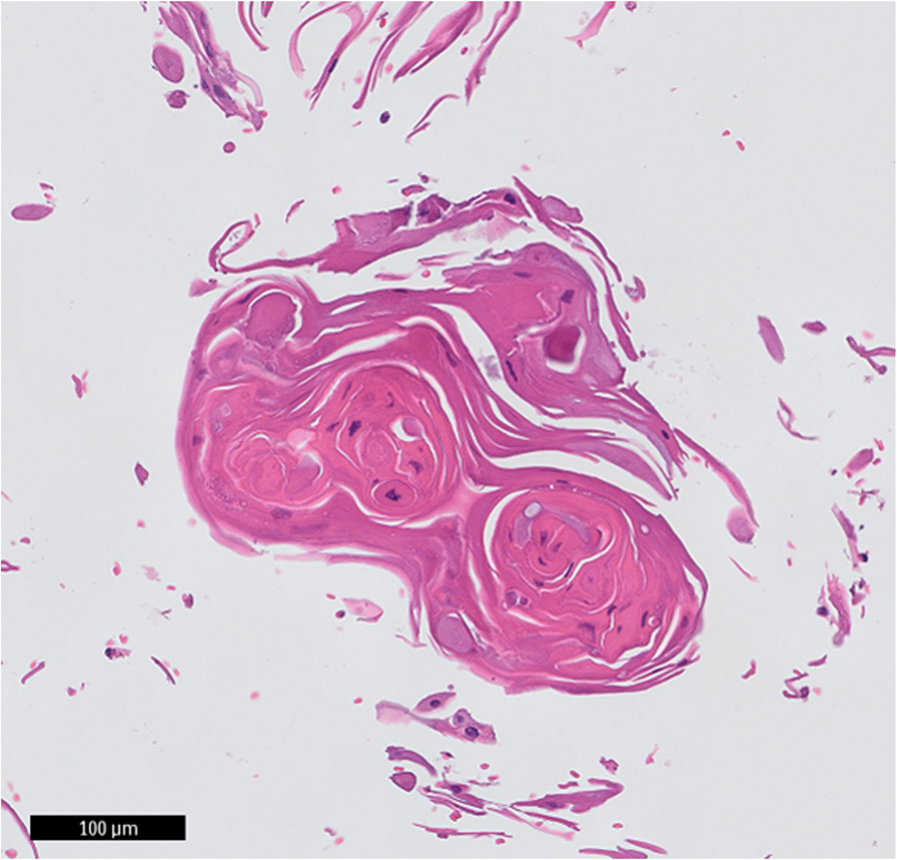
Lymph node metastasis of the SCC of the tongue in patient 1. HE staining of a fine needle aspirate (FNA) of a cervical lymph node using a standard embedding procedure of cytology material and histologic processing. Atypical squamous cells with (para-)keratotic horn were seen, indicative of metastasized SCC

After starting sorafenib treatment, the patient developed several skin lesions (for a summary see Table 1). Furthermore, within 2 months of beginning sorafenib therapy the patient developed left-sided tongue complaints, originally histologically diagnosed and treated as mucosal hyperplasia with Candida albicans infection. The symptoms persisted however and eventually led, 46 months after the initial complaints, to the diagnosis of a T2N2cM0 functional irresectable SCC of the tongue (*thyroid transcription factor* (TTF)-1 and thyroglobulin immunohistochemically negative; *BRAF* p.V600E negative) with ipsi-lateral lymph node metastasis (Fig. 1). Despite chemo-radiation therapy with 7 rounds of cisplatin, the patient died 5 months after diagnosis of the SSC.

**Table 1:** Summary of lesions seen in patient 1 after starting sorafenib treatment November 2007

Date	Lesion
February 2008	SCC on the back Candida infection of the tongue
April 2008	Reactive epithelial skin lesion on the back without obvious atypia Leukokeratosis of the tongue with atypia and inflammation
June 2008	Lesion with inverted follicular keratosis on the lower left leg
September 2009	Trichilemmoma of the nose
March 2010	Irritated verruca seborrhoica upper right leg
May 2010	Reactive epithelial hyperplasia of the tongue due to a candida infection
August 2011	Invasive squamous cell carcinoma of the tongue Multiple lymph node metastasis of the SCC in the neck region
December 2011	SCC lymph node metastasis in the left axilla

#### Patient 2

**Fig. 2:**
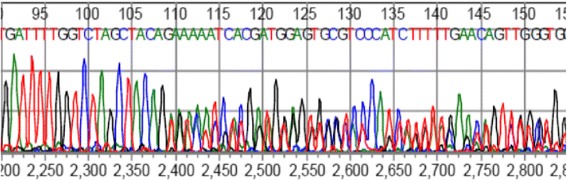
*BRAF* DNA sequencing. Identification of a rare *BRAF* mutation c.1799-1801het_delTGA

**Fig. 3:**
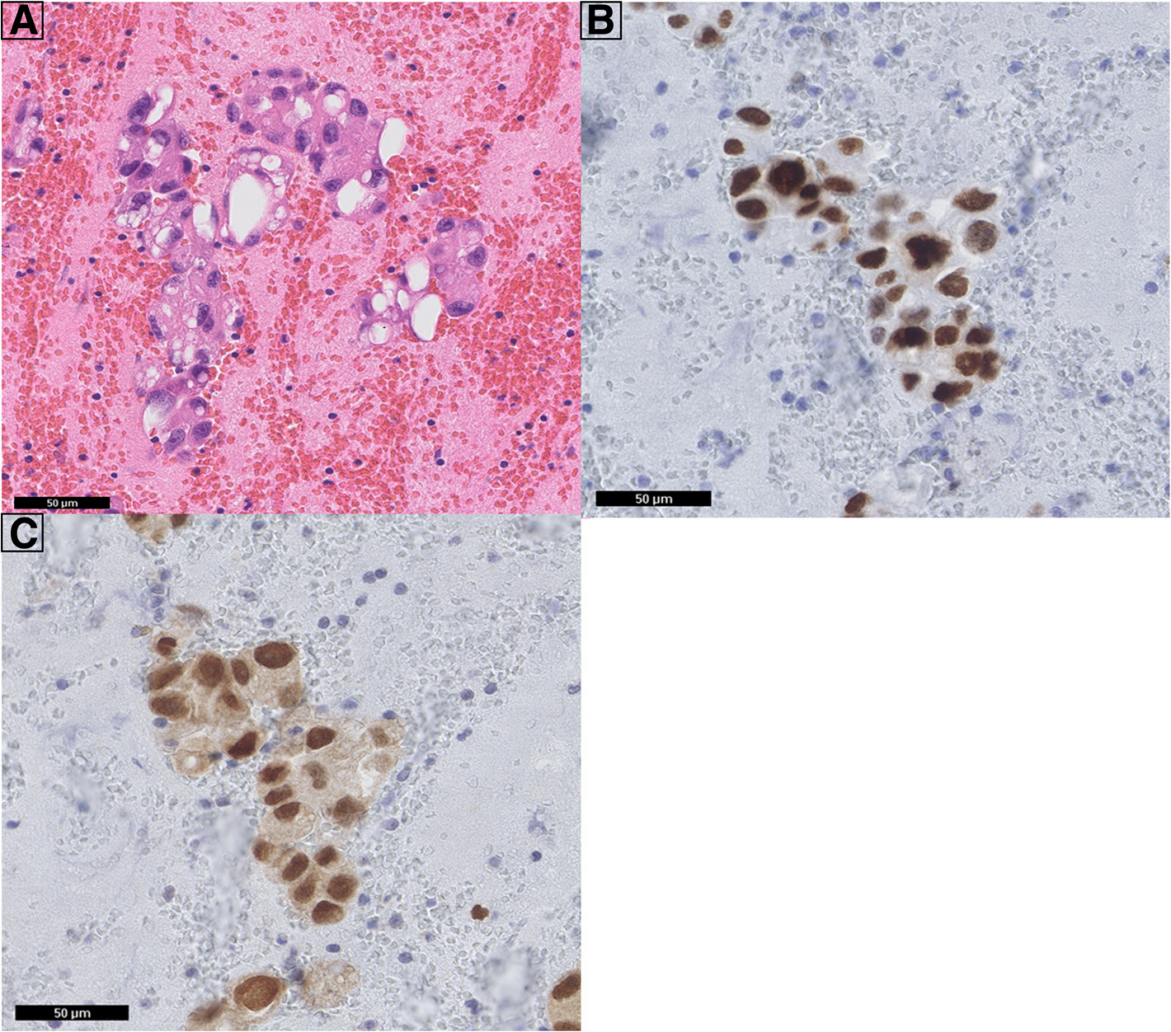
Pathological analysis of pleural effusion in patient 2. FNA of a right-sided pleural effusion after embedding and histological processing. **a** HE; **b**: TTF1 immunohistochemistry (IHC); **c**: PAX8 IHC

The second (male) patient was diagnosed with a T4N1Mx PTC without squamous metaplasia (positive for the rare *BRAF* mutation c.1799-1801het_delTGA, Fig. 2) at the age 67 [12–14]. He underwent a total thyroidectomy with a right-sided lymph node dissection, followed by RAI ablation therapy in 2001. In 2004 the patient had recurrent disease, and a bilateral para-tracheal lymph node dissection was performed. A year later he presented with local recurrent disease and the presence of multiple pulmonary metastases. A whole body scintigraphy after RAI therapy showed no RAI uptake, while thyroglobulin levels were 133 ug/l, thus demonstrating RAI refractory disease. In 2007, the patient was referred to our hospital for inclusion in a phase II trial and received sorafenib 400 mg twice daily [2, 10]. Despite initial stable disease, the patient became progressive under sorafenib after 19 months of therapy. The patient was subsequently enrolled in a clinical study (RAD001, www.clinicaltrials.gov CRAD001CNL08T) to determine the efficacy of everolimus in patients with progressive irresectable recurrent or metastatic differentiated, undifferentiated (anaplastic) and medullary thyroid carcinoma. The patient ceased everolimus therapy 18 months later due to progressive disease. A CT showed a new pulmonary lesion and a number of (non)target lesions according to RECIST 1.0 [11]. Since all conventional or study-based treatment options had been exhausted, the patient was referred back to his own hospital where he presented 2 months later with progressive dyspnea due to malignant pleural effusion. A CT showed multiple bilateral pulmonary metastases and a large right para-tracheal lesion. Surprisingly, right-sided pleural fluid cytology elsewhere revealed a primary adenocarcinoma of the lung. Immunohistochemistry of embedded cytological material showed TTF-1 and *cytokeratin* (CK) 7 positivity and absence of staining for thyroglobulin, *cluster of differentiation* (CD) 56 and CK20. Additional *paired box* (PAX) 8 and CK19 staining in our institute were however positive, indicative of metastatic PTC (Fig. 3). Molecular testing confirmed this diagnosis with the presence of the previous identified *BRAF* mutation. The patient died a month later due to disease progression.

#### Patient 3

**Fig. 4:**
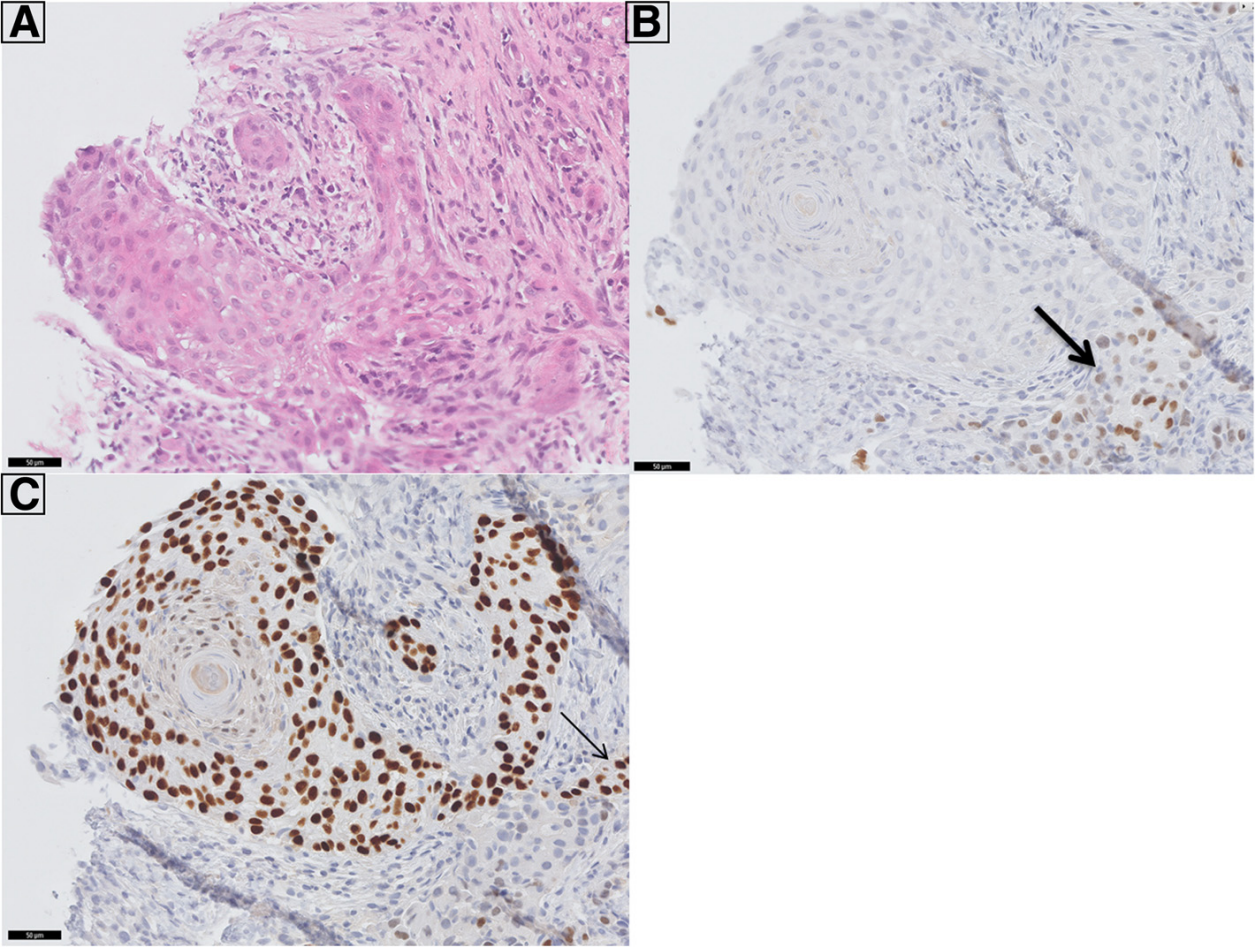
PTC recurrence in the laryngeal cricoid of patient 3. In a biopsy from the laryngeal cricoid solid sheets of tumor cells were seen with focal keratinisation. Panel **a** shows the HE stained tissue. Immunohistochemical analysis of TTF-1 (panel **b**) and P63 (Panel **c**) is shown. A large proportion of the cells stained positive for P63, supporting the squamous features. However, there was a fraction a cells that stained moderately positive for TTF-1 (thick arrow in panel **b**), partly overlapping with P63 positivity (thin arrow in panel **c**). Thyroglobulin also stained focally positive (not shown). Due to the TTF-1 and thyroglobulin positivity, we favoured the diagnosis of recurrent papillary thyroid cancer with remarkable squamous metaplasia over a primary laryngeal squamous carcinoma

A 60-year-old male was diagnosed with a T2N0M0 poorly differentiated PTC (*BRAF* c.1799 T > A; V600E mutation positive) in 2002. The patient was treated by total thyroidectomy with a left-sided lymph node dissection, followed by RAI ablation therapy. Three years later (2005) he presented with multiple pulmonary lesions, non-RAI avid on whole body scintigraphy after RAI therapy, and thyroglobulin levels of 127 ug/l. Due to disease progression in 2008 the patient was included in a phase II trial (and received 2 x 400 mg sorafenib daily) [2, 10]. In the first 6 months a partial response was achieved, which was ongoing until therapy discontinuation 32 months later due to complaints of diarrhea and hand-foot syndrome. A CT four months after therapy discontinuation still showed an ongoing partial response per RECIST 1.0 [11]. However, seven weeks later the patient presented with respiratory failure due to airway obstruction. A large tumor originating from the cricoid was visible on CT scan. Initial histological examination elsewhere concluded that the lesion was a SCC originating from the cricoid. Revision of the tumor showed a lesion with squamous differentiation (Fig. 4). Although P63 staining (indicative of squamous differentiation) was positive in a subset of cells, immunohistochemistry also showed TTF-1 and thyroglobulin to be focally positive, indicative of a PTC recurrence. Surprisingly, the *BRAF* mutation present in the primary tumor was not recovered from this tumor, possibly suggesting clonal divergence after sorafenib treatment. Given the fact that local treatment was not an option the patient received a tracheal stoma followed by radiotherapy. He died 8 months later due to tumor progression.

## Discussion

The cases described here present three interesting observations. Firstly, (although lacking proven causality) our demonstration of a primary tongue carcinoma that arose after long-term sorafenib treatment suggests that treatment with the multikinase inhibitor sorafenib may cause other effects additional to squamous skin lesions [3–8]. However, since patient 2 was subsequently treated with everolimus, a combined effect of sorafenib and everolimus cannot be ruled out. Secondly, we note that metaplastic changes or clonal divergence can result in the misdiagnosis of recurrences of thyroid cancer. Indeed, clonal divergence at the molecular level might be an aspect of PTC with *BRAF* c.1799 T > A; p.V600E mutations. Thirdly, we identified a rare *BRAF* mutation, c.1799-1801het_delTGA, which will be missed by the allele-specific assays currently in use in daily practice in many laboratories.

It has been proposed that *BRAF* kinase inhibitors can stimulate proliferation in cells lacking mutant *BRAF* via a paradoxical activation of RAF-MEK-ERK1/2 pathway signaling in the presence of upstream activation of RAS, thus possibly explaining the development of cutaneous SCC [3–8]. This hypothesis was supported by a study that analyzed oncogenic mutations in cutaneous SCCs in melanoma patients treated with the *BRAF* inhibitor vemurafenib. This study found that 13 out of 21 tumors harbored a RAS mutation [15]. Furthermore, there are several reports on patients treated with a *BRAF* inhibitor who developed secondary tumors. One case has been described of a patient on vemurafenib treatment for a melanoma who developed a RAS-mutant leukemia correlating with enhanced extracellular signal-regulated kinases (ERK) signaling [16]. Another study reports the development of four colonic adenomas, one hyperplastic colonic polyp and six gastric polyps in a patient also treated with vemurafenib for a melanoma. However, no evidence of RAS mutations was reported in any of the adenomas identified in this patient [17]. In addition, progression of a previously present KRAS mutated colon carcinoma due to stimulated ERK signaling was reported in a patient with a melanoma treated with dabrafenib [18]. A similar molecular explanation might be relevant to the cases we now present, although the presence of RAS mutations was not completely tested in the lesions with squamous metaplasia.

## Conclusions

In summary, we described three patients with advanced RAI refractory DTC and a history of sorafenib treatment who presented with a secondary non-cutaneous lesion. The first patient was diagnosed with a SCC of the tongue; the remaining two patients had recurrent disease with altered morphology and molecular biology suggesting clonal divergence, initial diagnosed as a primary adenocarcinoma and SCC, originating from the lung and cricoid respectively. Although the possibility that these tumors were already present in the form of micrometastases prior to sorafenib treatment cannot be excluded, these observations are quite remarkable. It is therefore important that a pathologist is familiar with the patient’s medical history and treatment and that the possibility of unusual presentation is kept in mind when considering secondary lesions.

### Consent

Written informed consent was obtained from the patients for publication of this case report and any accompanying images. A copy of the written consents is available for review by the Editor of this journal.

## Abbreviations

AE: Adverse event
BRAF: B-type Raf kinase
CD: Cluster of differentiation
CK: Cytokeratin
CT: Computed tomography
DTC: Differentiated thyroid carcinoma
EMA: European Medicines Agency
ERK: Extracellular signal-regulated kinases
FNA: Fine needle aspirate
HCC: Hepatocellular carcinoma
HE: Hematoxylin eosin
IHC: Immunohistochemistry
TKI: Tyrosine kinase inhibitor
PAX: Paired box
PTC: Papillary thyroid carcinoma
qPCR: Quantitative real-time PCR
RAI: Radioactive iodine
RCC: Renal cell carcinoma
RECIST: Response evaluation criteria in solid tumours
RET: Rearranged during transfection
SCC: Squamous cell carcinoma
TTF: Thyroid transcription factor
VEGFR: Vascular endothelial growth factor receptor.
